# Potent Ant Deterrents Emitted from Nematode-Infected Insect Cadavers

**DOI:** 10.1007/s10886-021-01320-8

**Published:** 2021-11-05

**Authors:** Geoffrey Jaffuel, Sribala Krishnamani, Ricardo A. R. Machado, Raquel Campos-Herrera, Ted C. J. Turlings

**Affiliations:** 1 FARCE Laboratory, Institute of Biology, University of Neuchâtel, Rue Emile-Argand 11, 2000 Neuchâtel, Switzerland; 2 Institute of Plant Sciences, University of Bern, Altenbergrain 21, 3013 Bern, Switzerland; 3 Experimental Biology Research Group, Institute of Biology, University of Neuchâtel, Rue Emile-Argand 11, 2000 Neuchâtel, Switzerland; 4 Instituto de Ciencias de la Vid y del Vino, CSIC-Universidad de La Rioja-Gobierno de La Rioja, Av. Madre de Dios 53, 26007 Logroño, Spain

**Keywords:** *Lasius niger*, *Steinernema feltiae*, Hexadecanal, 2-heptadecanone, Repellents

## Abstract

Most known species of entomopathogenic nematodes (EPNs) are generalist obligate parasites of insects. They kill their hosts within days after infection and mortality is mainly caused by toxins produced by bacteria that co-infect the hosts and serve as food for the nematodes. EPNs can infect a very broad spectrum of insects and these insects can therefore be expected to have evolved strategies to avoid infection. Indeed, ants are known to avoid feeding on EPN-infected insect cadavers, most likely because they are repelled by semiochemicals that emanate from the cadavers. The source and nature of these repellents are so far unknown. In a series of behavioral and chemical analytical experiments we identified hexadecanal and 2-heptadecanone as two compounds that are emitted by insect larva that are infected by the EPN *Steinernema feltiae*, but not by uninfected larvae. When spiking honey water with the two semiochemicals, they were confirmed to be highly deterrent to the ant *Lasius niger*. The environmentally benign hexadecanal and 2-heptadecanone could be employed to ward off ants and possibly other pests. Additional experiments are needed to fully determine their application potential.

## Introduction

Nematodes are extremely diverse and occupy nearly every possible ecological niche, including a wide diversity of parasitic niches (Weischer and Brown 2000). Amongst the vast variety of parasitic nematodes, some have evolved a close association with insect pathogenic bacteria and together with these bacteria act as parasites of insects. These nematodes are referred to as ‘entomopathogenic nematodes’ (EPNs) (Steiner 1923; Poinar and Grewal 2012). The most commonly studied EPN species belong to the families Steinernematidae and Heterorhabditidae, and are lethal parasites of many epigeic and subterranean insects (Kaya and Gaugler 1993; Hominick et al. 1996; Dillman et al. 2012). The genera *Steinernema* and *Heterorhabditis* are symbiotically associated with bacteria of the genera *Xenorhabdus* and *Photorhabdus*, respectively (Goodrich-Blair and Clarke 2007). In the genus *Steinernema*, the bacteria are carried monoxenically in a specific vesicle by the infective juvenile (IJ), which is the non-feeding, free living stage that survives outside of the host. In *Heterorhabditis*, the bacteria are located in the posterior part of the intestine of the IJ’s (Goodrich-Blair and Clarke 2007). After having located an insect host, an IJ penetrates the insect body cavity via natural openings (mouth, anus, spiracles) and thinner parts of the cuticle. Once inside the body cavity, the symbiotic bacteria are released and proliferate, typically releasing a toxin that kills the host within two to five days (Akhurst 1982; Boemare and Akhurst 1988; Kaya and Gaugler 1993; Dillman et al. 2012; Stock 2015). Inside the host, the nematodes produce several generations of obligate parasites, and after 7-14 days a final generation of new IJs will leave the host cadaver by the thousands in search of new hosts (Dillman et al. 2012; Stock 2015).

While nematodes complete the parasitic stages inside the host cadaver, it is essential that the cadavers remain intact, because these stages cannot survive outside of the cadaver (Baur et al. 1998). For the successful infection and preservation of the cadaver, the nematodes rely heavily on the bacteria, which, in turn, benefit from the symbiotic association via the effective transmission by the EPN (Forst and Clarke 2002). The bacteria also help to protect the cadavers from competing nematodes, from bacterial and fungal growth (Ehlers 1996), as well as from insect scavengers (Baur et al. 1998; Zhou et al. 2002; Gulcu et al. 2012). The latter protection may involve the production of repellent compounds by the endosymbiotic bacteria while inside the insect cadaver (Webster 2002). Previous studies confirm that there are specific olfactory cues associated with EPN-infected cadavers. For instance, EPN-infected cadavers were found to attract larvae of the beetle *Diabrotica virgifera*, which can serve as hosts (Zhang et al. 2019), or *Sancassania polyphyllae*, a mite that feeds on dead insects (Cakmak et al. 2013), but also to repel IJs of con- and heterospecific species of EPN (Fu et al. 2021). A first study on scavengers conducted in California showed that the Argentine ant, *Linepithema humile* (Mayr), as well as other species of ants do scavenge EPN-infected insect cadavers, but not if they contained specific bacterial strains (Baur et al. 1998). They found that insects killed by an injection of the bacterium *Photorhabdus luminescens* (Thomas and Poinar) from *Heterorhabditis bacteriophora* Poinar, were very much avoided by the ants, contrary to insects killed by an injection of *Xenorhabdus nematophilus* (Poinar and Thomas) from *Steinernema carpocapsae* (Weiser). Additional research confirmed that the symbiotic bacteria isolated from certain species of EPNs produce compound(s) that deters ants and protect the cadavers from being eaten. The effectiveness of these “ant deterrent factor(s)” (ADF) was different for different ant species, and dependent on the strain, form, and age of the bacteria Zhou et al. (2002). Contrary to Baur et al. (1998), Zhou et al. (2002) found no difference in the deterrent effect between *Xenorhabdus* and *Photorhabdus* bacterial strains. A more recent study with the ant *Lepisiota frauenfeldi*, the cricket *Gryllus bimaculatus*, the wasps *Vespa orientalis* and *Paravespula* sp., and the calliphorid fly *Chrysomya albiceps* showed that all of these potential scavengers of EPN-infected cadavers are deterred by chemical compound(s) produced by the symbiotic bacteria (Gulcu et al. 2012). The identity of these compounds, that help to prevent scavengers disrupting the lifecycles of the nematodes as well as the bacteria, has so far been elusive.

The aim of the current study was to identify repellents or deterrents emitted by nematode-killed cadavers using volatile analysis and behavioral assays. We chose to work with the EPN *Steinernema feltiae* because *S. feltiae*-killed larvae are known to have high ant deterrent activity (Baur et al. 1998; Gulcu et al. 2012). Dip extracts were obtained by dipping living *Galleria mellonella (L)* larvae, as well as freeze-killed and *S. feltiae*-killed *G. mellonella* larvae into solvents. By resuspending the extracts in drops of diluted honey, we tested their deterrence to ants (*L. niger*) in two-choice and four-choice bioassays. *L. niger* is a widespread ant species that feeds on honeydew, living or dead insects, as well as on plants and fungi. We then conducted chemical analyses to compare volatile compounds in the dip extracts in order to identify candidate repellent/deterrent compound(s). The repellence or deterrence of these candidate compounds was then tested and confirmed in additional choice assays with honey-water drops.

## Materials and Methods

### Nematode Cultures and Ant Colonies

*Steinernema feltiae* Filipjev was obtained from Andermatt Biocontrol (Grossdietwil, Switzerland). The nematode colony was maintained and cultured using last instar larvae of *Galleria mellonella* (L.) at room temperature (23/24 °C) as previously described (Kaya and Stock 1997). The nematode-infected larvae were placed on a White trap and emerging infective juveniles (IJs) were collected from the water (White 1927; Weischer and Brown 2000). The IJs were stored at 15 °C in culture flasks.

Laboratory grown colonies of the ant *L. niger* were obtained from the University of Lausanne (n = 14). Each colony consisted of workers (50-500), a queen, and their brood. The colonies were maintained with an artificial diet that was prepared using a mixture of 4 eggs, honey (500 g), water (32 cl), and agar (24 g). The colonies were also provided with a tube containing a 10% honey-water mixture and *Tenebrio molitor* (mealworms). The colonies were fed every week.

### Solvent Dip Extraction and Sample Preparation

Freeze-killed (−80 °C), living, or EPN-killed (4 days after infection) *G. mellonella* larvae were dipped in 400 μl of hexane for 30 s (n = 15). In preliminary experiments we also used methanol, dichloromethane, but found that hexane was most effective in extracting the compounds of interest. The hexane dip extracts were evaporated under a nitrogen gas flow in a fume hood at room temperature, resuspended in 100 μl of hexane, and stored in 1 ml glass vials (BGB Analytik AG, Böckten, Switzerland). For the purpose of GC/MS analyses, 100 μl of each dip extract was transferred into a 200 μl insert and 10 μl of internal standard solution containing nonyl-acetate (20 ng/μl in hexane) and eucalyptol (20 ng/μl in hexane) were added. Vials were stored at −80 °C prior to analyses. For the purpose of bioassays, the original dip extracts were left to evaporate under nitrogen gas in a fume hood at room temperature and the dip extracts were resuspended in 100 μl of MilliQ water. As a control, 100 μl of hexane was added to a vial, evaporated, and resuspended in 100 μl of MilliQ water. Vials were stored at −80 °C prior to bioassays.

### Two-Choice and Four-Choice Honey-Water Bioassays

We first tested the effects of the different dip extracts on ant preferences in a dual-choice bioassay. Before using it in bioassays, each type of hexane dip extract was evaporated under a nitrogen flow and the residue was resuspended in 100 μl of water. This mixture was mixed with 100 μl of a solution of water and honey (10%) in an Eppendorf tube lid, diluting the active substances by a factor of two. Control solutions were obtained by evaporating 400 μl of hexane, after which 100 μl of MilliQ water was added to 100 μl of honey-water. Twelve workers of *L. niger* were placed in a petri dish (100 × 25 mm) containing two Eppendorf tube lids, one with the dip extract and the other with the control solution. The bioassays were monitored by video over a period of 4 h using the iSpy software (https://www.ispyconnect.com/). The position of the ants in relation to the Eppendorf tube was noted every minute. The bioassay was carried out with 8 randomly selected *L. niger* colonies and was replicated 3 times within each colony.

A similar series of four-choice bioassays were conducted, in which the ants were offered all of the 3 types of dip extracts and the control simultaneously in the same petri dish. This assay was replicated 2 times for 8 randomly selected ant colonies.

### Chemical Profile and Identification

The different dip extracts (n = 15) were analyzed using a gas chromatograph coupled with a quadrupole mass spectrometer detector (6890 GC coupled to a 5973 quadrupole MS, Agilent, Santa Clara, CA). A 2 μl aliquot of each sample was injected in pulsed splitless mode into an Agilent HP-5MS column (30 m length × 250 μm diameter and 0.25 μm film thickness). After injection, temperature was maintained at 69 °C for 3.5 min, increased to 100 °C at a rate of 8 °C per min and subsequently to 230 °C at a rate of 5 °C per min followed by a post run of 3 min at 250 °C. Transfer line temperature was set to 280 °C, electron ionization to 70 eV. Helium was the carrier gas and was kept at a constant flow of 0.9 ml/min. Compounds were identified by comparing their mass spectra with those from the NIST MASS spectral library (D.05.02, U.S. Department of Commerce) (Johnson 2016) and by calculating Kovats retention indices (Kovats 1958) after injection of a solution containing C8-C40 alkanes. Quantifications of the compounds of interest were obtained based on the peak areas of these compounds compared to the peak areas of the internal standards (Turlings et al. 1998).

### Individual Two-Choice and Mixed Bioassays of Candidate Compounds

We tested the deterrence of two of the identified compounds (2-heptadecanone and hexadecanal) that were consistently and exclusively found in the dip extracts of EPN-killed *G. mellonella* larvae. Both compounds were obtained from TCI chemicals GmbH (Eschborn, Germany). The compounds were diluted in hexane to the concentrations measured in the GC/MS analyses (0.48 ng/μl and 1.4 ng/μl, for the hexadecanal and the 2-heptadecanone respectively). The hexane was evaporated under a nitrogen flow and the compounds’ residues were resuspended in 100 μl of water. The 100 μl of aqueous mixture containing the compound was mixed with 100 μl of a solution of water and honey (10%) in an Eppendorf tube lid. The bioassays were performed similarly to the dual-choice test bioassay used with the original dip extracts. Briefly, twelve workers of *L. niger* were added to a petri dish (100 × 25 mm) containing an Eppendorf tube lid with 100 μl honey (10%) water and 100 μl of one of the compounds and another lid with only 100 μl of honey-water as control. The bioassay was carried with 5 randomly selected *L. niger* colonies and replicated 2 times for each colony. Ant behavior was monitored by video over a period of 4 h using iSpy software. In additional series of assays, we used honey-water with a mixture of both compounds (at the same concentrations) next to control honey-water.

### Bioassays Measurement Criteria

**Fig. 1:**
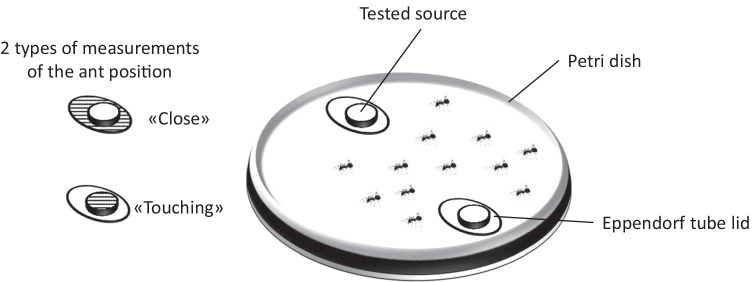
Schematic drawing of the ant deterrence bioassay. The hatched parts of the assay lids indicate where ant presence was recorded

The position of the ants on the Eppendorf lids was recorded every minute as “touching” and “close”. The “touching” measurements were recorded when the ants were in the center part of a lid where they would feed on the samples. The “close” measurements were noted when the ants were in outer part of an Eppendorf lid (Fig. 1).

### Statistical Analyses

Statistics were carried using R (R Development Core Team 2008). Data were analyzed using an “Exact Wilcoxon-Pratt Signed-Rank Test” from the package “coin”. This test allows analyzing non-parametric data as well as to cope with repeated measurement. When necessary, p-values were corrected by the Benjamini-Hochberg procedure (Benjamini and Hochberg 1995).

## Results

### Bioassays with Dip Extracts

**Fig. 2:**
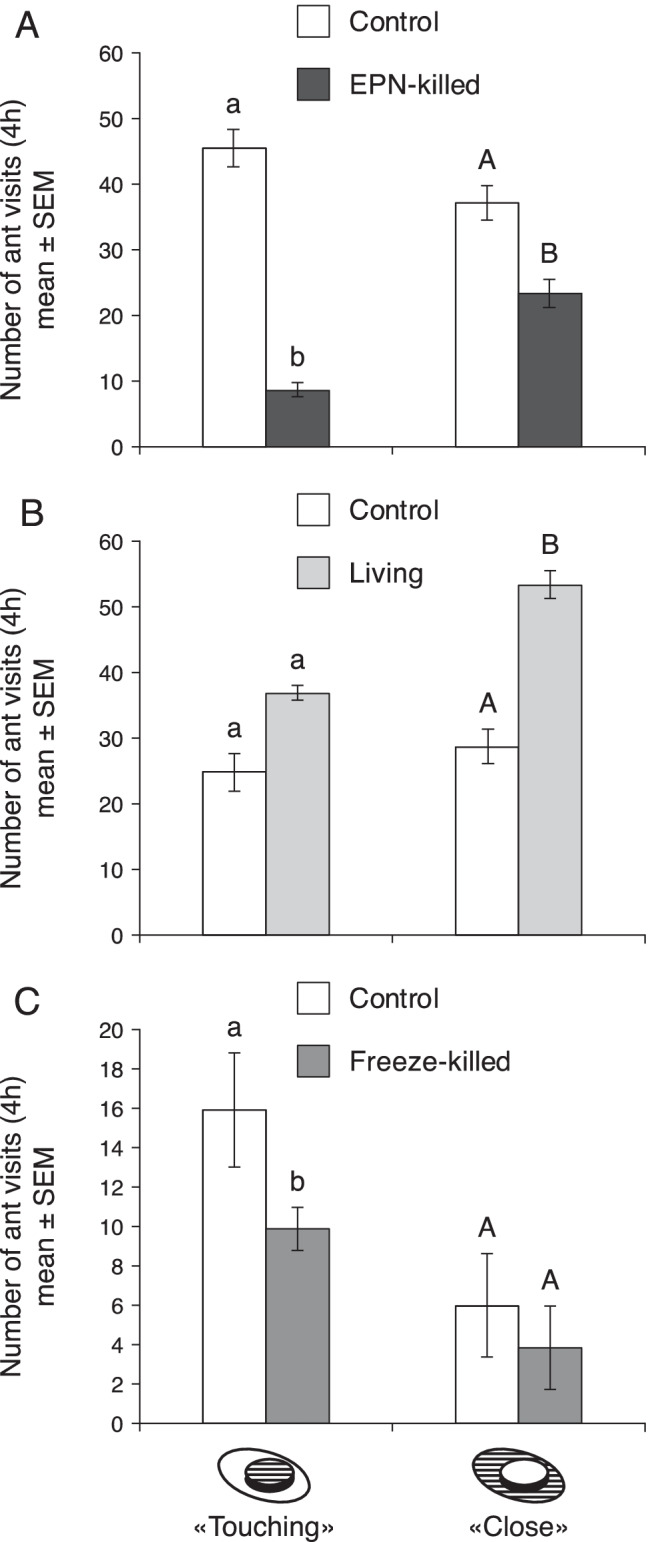
Mean number of ant visits during 4 h to A: honey water with solvent (control) and spiked with dip extracts from EPN-killed larvae, B: honey water with solvent (control) and spiked with dip extracts from living larvae and C: honey water with solvent (control) and spiked with dip extracts from freeze-killed larvae. The hatched parts of the assay lids indicate where ant presence was recorded. Bars represent mean ± SEM. Means with different letters are significantly different (p < 0.05)

Two-choice bioassay: More ants visited the Eppendorf tube with the control sugar solution as compared to the suspension spiked with the dip extract of EPN-killed larvae. For the “touching” measurement, the ants visited the control lids 5.3x more often than the lids with the dip extract of EPN-killed larvae (Z = 11.9945, p value <0.001, Fig. 2A). No significant difference in the frequency of visits was observed between dip extracts from living larvae and the honey water control (Z = 0.3202, p value = 0.7527, Fig. 2B). Controls were visited 1.6x more often than dip extracts from freeze-killed larvae (Z = 2.9678, p value <0.01, Fig. 2C). For the “close” measurement, the ants visited lids with the dip extracts of living larvae 1.8x more often than the controls (Z = −3.915, p value <0.001, Fig. 2B). The controls were visited as often as the dip extracts from freeze-killed larvae (Z = 1.4811, p value = 0.1259, Fig. 2C) and 1.6x more often than the dip extracts from EPN-killed larvae (Z = 5.1109, p value <0.001, Fig. 2A).

**Fig. 3:**
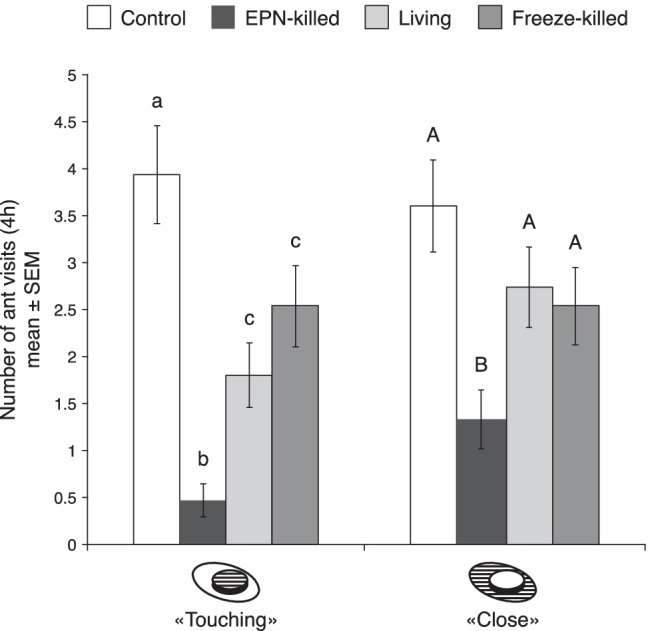
Mean number of ant visits during 4 h to honey water with solvent (control) and honey water spiked with dip extracts from living, freeze-killed and EPN-killed larvae. The hatched parts of the assay lids indicate where ant presence was recorded. Bars represent mean ± SEM. Means with different letters are significantly different (p < 0.05)

The four-choice bioassays confirmed the repellency of the EPN-killed larvae. For the “touching” measurements, there was a significant difference in the mean number of ant visits among the four treatments. The ants preferred to visit (“touching”) the control lids, whereas the lids with dip extracts from EPN-killed larvae were by far the least preferred by the ants. The mean number of ants that visited the controls was approximately 2x higher than lids with dip extracts from living larvae (Z = −3.3632, p value <0.01), 1.5x higher than with freeze-killed dip extracts (Z = −2.2668, p value = 0.03) and 8.4x higher than the dip extract from EPN-killed larvae (Z = −6.326, p value <0.001). For the “close” measurements there was no significant difference of ant visits among the control treatment and dip extracts from living and freeze-killed larvae, but the ants significantly preferred these three treatments over the dip extracts from EPN-killed larvae. No significant difference in the frequency of “close” visits was observed between dip extracts from living larvae and freeze-killed larvae as compared to the honey water control (Z = −1.3338, p value = 0.26, Z = −1.6865, p value = 0.16, respectively). The mean number of ants that approached (“close”) the controls was approximately 2.7x higher than that approaching the dip extracts from EPN-killed larvae (Z = −4.0947, p value <0.001; Fig. 3).

### GC-MS Analysis

**Fig. 4:**
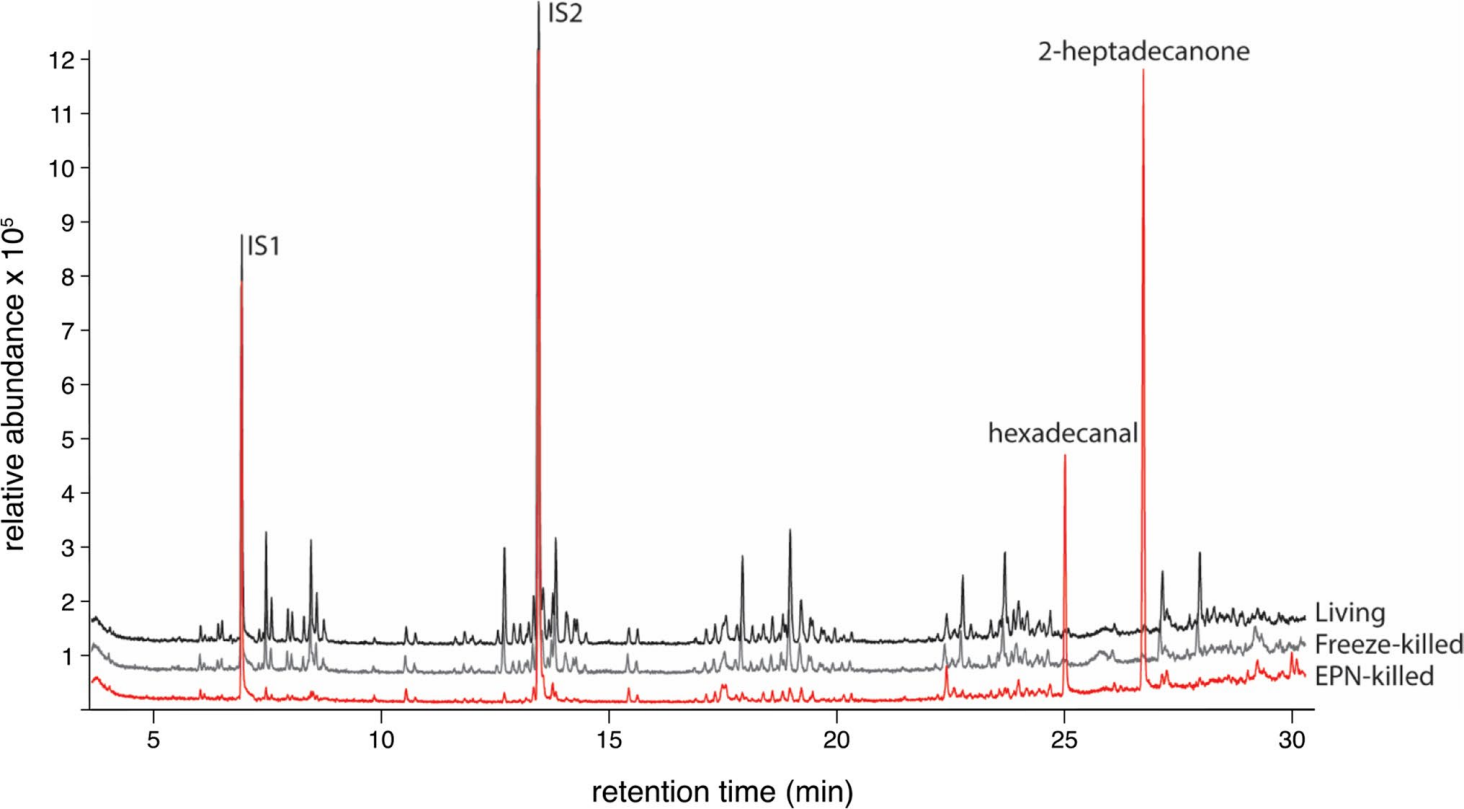
GC-MS chromatogram of dip extracts from living, freeze-killed and EPN-killed larvae, with hexadecanal and 2-heptadecanone only found in nematode dip extract samples. IS: Internal standard

Two distinctive peaks were present in large quantities in all of the samples collected from EPN-killed larvae. Ions characteristic for the two compounds were not detected in the dip extracts of freeze-killed and living larvae (Fig. 4). The corresponding compounds were identified as hexadecanal and 2-heptadecanone. Using calibration curves, their average quantities in each dip extract were calculated as 0.48 ng/μl for the hexadecanal and 1.4 ng/μl for 2-heptadecanone.

### Bioassays with Hexadecanal and 2-Heptadecanone

**Fig. 5:**
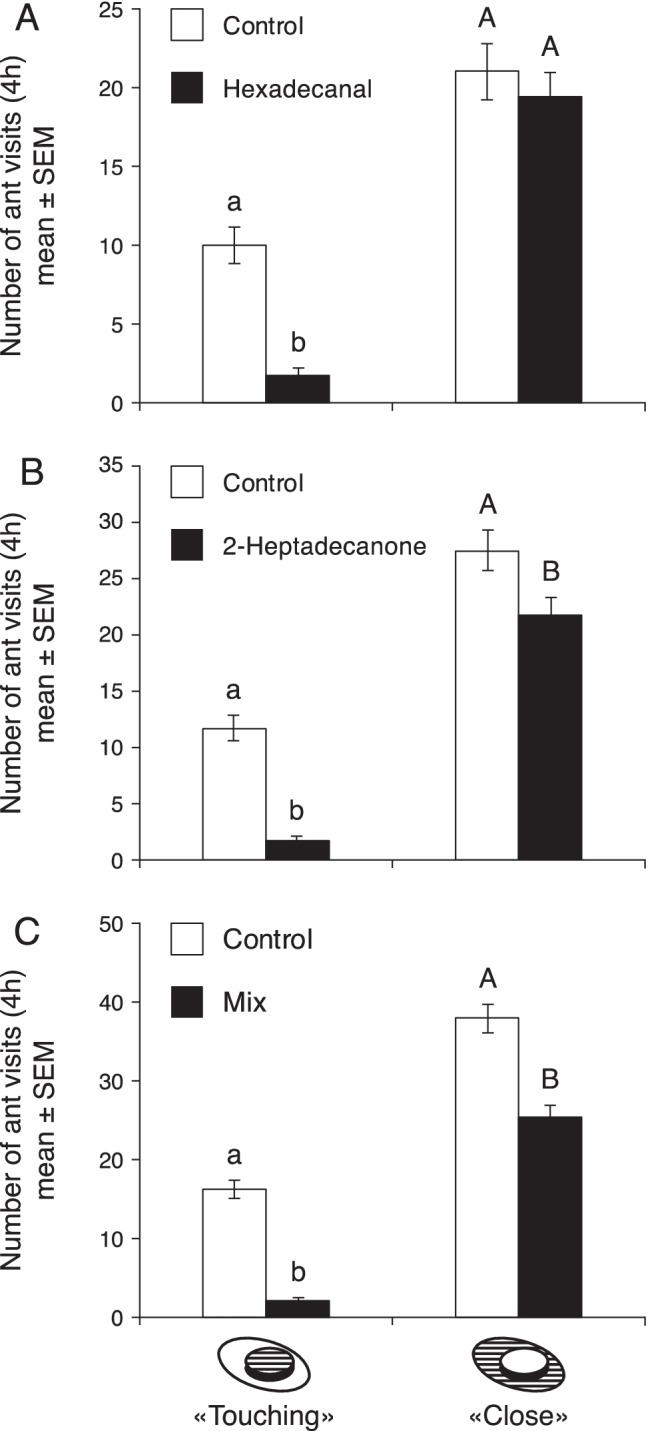
Mean number of ant visits over 4 h to A: control solution and hexadecanal sample, B: control solution and 2-heptadecanone, C: control solution and a mix of hexadecanal and 2-heptadecanone sample. The hatched parts of the assay lids indicate where ant presence was recorded. Bars represent mean ± SEM. Means denoted by different letters are significantly different (p < 0.05)

Bioassays were performed similarly to the two-choice test bioassay performed with the original dip extracts. The ants visited (“touching”) the controls 5.8x more often than the hexadecanal-spiked suspension (Z = 7.808, p value <0.001), but they approached (“close”) the controls at a similar rate as the hexadecanal suspension (Z = 0.95716, p value = 0.3479; Fig. 5A). Control solutions were visited (“touching”) 6.8x times more often than suspensions spiked with 2-heptadecanone (Z = 8.5606, p value <0.001). The ants also approached (“close”) the controls (1.2x) more than the 2-heptadecanone-spiked suspension (Z = 2.37, p value <0.05, Fig. 5B). When we combined hexadecanal and 2-heptadecanone, the ants visited (“touching”) the controls 8x times more often (Z = 9.8606, p value <0.001, Fig. 5C), and approached (“close”) the controls 1.5x times more often (Z = 5.8234, p value <0.001, Fig. 5C).

## Discussion

We found that hexane dip extracts of *G. mellonella* larvae killed by EPN repel the ant *L. niger*. Specifically, considerably fewer ants would visit honey-water drops that were spiked with these dip extracts than control (unspiked) drops or drops that were spiked with the dip extracts of living or freeze-killed larvae. Using GC/MS analyses of the dip extracts, we identified two compounds, hexadecanal and 2-heptadecanone, that were exclusively found in the dip extracts of EPN*-*killed *G. mellonella* larvae, and not in the other dip extracts. Feeding assays with pure versions of these compounds at the same concentrations that were found in the dip extracts confirmed their repellent effects on the ants. Combined, the compounds were slightly more effective than when the honey-water only contained one of them. The larval dip extracts as well as the two compounds were more active when the ants were very close to the bait (“touching”) and therefore it might be better to consider their effect as deterrent rather than repellent, which is in accordance with previous studies with EPN-infected insect cadavers (Baur et al. 1998; Zhou et al. 2002; Gulcu et al. 2012). It should be noted that the experiments with the original dip extracts also indicated a repellent effect and that other, more volatile compounds may also contribute to the avoidance of EPN-infected larvae by the ants.

The nematode used in this study was *S. feltiae*, which carries and relies on the bacterium *Xenorhabdus bovienii* for successful infections. It is assumed that the bacteria are the source of the deterrents (Zhou et al. 2002; Gulcu et al. 2012), but this remains to be determined for our study system. As yet, it is also not known if insects that are infected by other EPN and associated bacteria produce or enhance the same or similar deterrents, and if other ant species and other arthropods are equally deterred by these compounds.

It should be noted that, in ant assays, we spiked the honey-water with half of the equivalent of what was dip extracted from one nematode-infected larva. These minute amounts (around 0.5 ng/μl) had already a clear deterrent effect. Commercially available repellents/deterrents are usual applied in much higher concentrations, which implies that hexadecanal and 2-heptadecanone may have potential for commercial application. To determine their full potential, further tests with additional target insects, different concentrations, application methods and formulations are needed.

## Data Availability

The authors declare that all the data is available to third parties.

## Code Availability

Only open accessed codes were used during the study.
